# Emerging uncertainty on the anti-aging potential of metformin

**DOI:** 10.1016/j.arr.2025.102817

**Published:** 2025-06-27

**Authors:** Matthew Thomas Keys, Jesper Hallas, Richard A. Miller, Samy Suissa, Kaare Christensen

**Affiliations:** a Epidemiology, Biostatistics, and Biodemography, Department of Public Health, University of Southern Denmark, Odense, Denmark; b Clinical Pharmacology and Pharmacy, Department of Public Health, University of Southern Denmark, Odense, Denmark; c Department of Pathology, University of Michigan, Ann Arbor, MI, USA; d Departments of Epidemiology and Biostatistics, and Medicine, McGill University, Montreal, Canada; e Danish Ageing Research Centre, University of Southern Denmark, Odense, Denmark

**Keywords:** Metformin, Anti-aging, Type 2 diabetes, Longevity, Lifespan, Geroscience

## Abstract

Metformin is the most commonly prescribed glucose-lowering agent worldwide for the treatment of type II diabetes. Due to evidence of improvements in healthspan and lifespan in model organisms, and mechanistic data relevant to the hallmarks of aging, it has been considered a promising candidate in the search for pharmacological interventions that may attenuate the ageing process in humans. Various epidemiological studies have been influential in generating support for this hypothesis. These include pronounced anticancer and cardioprotective benefits compared to other antidiabetic treatments, and an observation of metformin use in type II diabetes being associated with better survival than that of the general population. Here we discuss recent developments in the evidence underlying the rationale for using metformin to target ageing. We describe the methodological limitations of some of the early and most influential findings and critically assess their scientific follow-up, including replication attempts of key experimental and observational findings, and a range of clinical trials of metformin in individuals without type II diabetes. These developments generally illustrate an emerging uncertainty in the anti-aging potential of metformin.

## 1. Introduction

Metformin is an oral hypoglycaemic agent of the biguanide class, developed in the 1940s, and first used to treat the symptoms of adult-onset diabetes in 1957 (Bailey and Turner, 1996). The results of later clinical trials in the late 1990s transformed metformin into the preferred first-line therapy for the treatment of type II diabetes worldwide - a position which it retains to this day (Bailey, 2017). Metformin’s complex mechanism of action has interested researchers from a range of disciplines and inspired wide-ranging investigations for its use and repurposing outside of type II diabetes (Viollet et al., 2011; Foretz et al., 2014). More recently, metformin has emerged as a promising candidate in the search for pharmacological interventions that may attenuate the aging process in humans (Campisi et al., 2019; Partridge et al., 2020). Such data have been compelling for the geroscience initiative, where the aim is to understand how aging facilitates chronic disease (Kennedy et al., 2014). A key observation here is that interventions which prolong lifespan also have the effect of delaying or preventing a range of age-related diseases (Longo et al., 2015).

Compared to validating new compounds, repurposing existing medicines as anti-aging drugs may provide faster progress due to existing safety data and lower regulatory barriers, and do so at a fraction of the cost (Kulkarni et al., 2022; Rolland et al., 2023). The most serious attempt to advance this initiative is with a proposal for an FDA-approved phase III randomised placebo-controlled clinical trial of metformin in individuals without type II diabetes, with a composite primary endpoint comprised of mortality, major cardiovascular events, incident cancers, and cognitive impairment (Barzilai et al., 2016; Justice et al., 2018). There are considerable expectations for such an undertaking, given the perceived strength of the evidence that informs its rationale, and the far-reaching consequences if successful (Hall, 2015). These include reducing age-related morbidity for hundreds of millions of people worldwide and shifting the paradigm for how regulatory agencies evaluate drugs for multimorbidity and aging (Barzilai et al., 2018). In this review, we describe the evolution of the experimental and human evidence relevant to metformin’s putative anti-aging effects, including the early and most influential lines of research supporting its rationale, as well as a critical assessment of their contemporary scientific follow-up.

## 2. Early evidence supporting the anti-aging potential of metformin

### 2.1. Effects on biology of aging and lifespan in experimental models

Metformin has a complex mechanism of action that may be relevant to the treatment or prevention of a range of disease states beyond hyperglycaemia. Importantly, many of these mechanisms are upstream or downstream to the hallmarks of aging (López-Otín et al., 2013, 2023). These are biomarkers that associate with increasing age and chronic disease risk, and modulate age-associated deterioration under experimental manipulation (López-Otín et al., 2013, 2023). Notably, metformin is associated with activation of adenosine monophosphate-activated protein kinase (AMPK), although it is unclear if this is the primary target (Zhou et al., 2001; Ferrannini, 2014; Foretz et al., 2019). By 2013, there was an increasing body of evidence suggesting metformin could increase lifespan in experimental rodent models (Martin-Montalvo et al., 2013; Anisimov et al., 2008, 2011, 2005). These findings merged with the consensus from basic science, and provided renewed optimism for the clinical potential of these effects (Anisimov, 2013; de Cabo et al., 2014; Kennedy and Pennypacker, 2014). In the most widely discussed of these studies, Martin-Montalvo et al. (2013) administered metformin at a dose of 0.1 % of diet by weight to C57BL/6 inbred male mice in mid-life, and reported an increase in mean lifespan of 5.8 % and improvements in several measures of healthspan. Metformin produced transcriptomic changes similar to those induced by caloric restriction, increased antioxidant defences, preserved mitochondrial function, and inhibited chronic inflammation, all of which may have been involved in the mechanisms underlying its reported effects on lifespan (Martin-Montalvo et al., 2013).

### 2.2. Efficacy as a first-line treatment for type II diabetes

Metformin has featured in several landmark trials for its use in the treatment of type II diabetes. A subgroup analysis of the randomized UK Prospective Diabetes Study (UKPDS) showed that initiating intensive glucose-control with metformin reduced the risk of diabetes-related events (relative reduction 32 %), diabetes-related death (42 %) and all-cause mortality (36 %), compared to dietary treatment alone (UKPDS Research Group, 1998). Long-term follow-up showed that such cardiovascular effects persisted over 15 years, suggesting a legacy effect of early intervention in the treatment of hyperglycaemia (Holman et al., 2008; Chalmers and Cooper, 2008). Notably, metformin’s effects were larger than that of other treatment arms of the study, despite equivalent glycaemic control (UKPDS Research Group, 1998; Holman et al., 2008). The HOME trial demonstrated that intensive therapy with insulin and metformin together was associated with lower risk for major cardiovascular events compared to insulin monotherapy (Kooy et al., 2009). The SPREAD-DIMCAD trial showed that metformin was associated with a lower risk of major cardiovascular events in direct comparison to the sulfonylurea glipizide (Hong et al., 2013). However, due to the active comparator design, this trial could not determine whether metformin is beneficial, or glipizide is harmful, given long-standing concerns about the cardiovascular safety of sulfonylureas (Monami et al., 2013a; Bain et al., 2017). The findings of UKPDS, HOME and SPREAD-DIMCAD have often been interpreted as suggesting that metformin has cardiovascular effects which are independent of its glycaemic mechanisms (Inzucchi, 2017).

### 2.3. Protection against age-related diseases in observational studies

Metformin and its associations with disease risk and mortality have been assessed in a large number of observational studies. The vast majority of these were conducted on individuals with type II diabetes, in which metformin was compared to other glucose-lowering drugs. One of the most prominent lines of research in this area pertains to evidence of its protective effects against cancer, with early meta-analyses reporting risk reductions in both cancer incidence and mortality associated with metformin of approximately 30–40 % (Decensi et al., 2010; Zhang et al., 2013; Noto et al., 2012). Mortality benefits have also been observed when metformin was initiated after cancer had been diagnosed, indicating its potential as an adjuvant therapy (Coyle et al., 2016). Similar findings have been reported for various manifestations of cardiovascular disease, where the magnitude of benefit in observational studies has also often been larger than that reported in clinical trials (Eurich et al., 2013; Pladevall et al., 2016; Evans et al., 2006; Pantalone et al., 2012; Han et al., 2019). One of the most highly cited meta-analyses on metformin and aging in observational studies by Campbell et al. (2017) suggested consistently protective effects against a wide range of age-related conditions, including dementia (Campbell et al., 2017). The magnitude of metformin’s observational associations with health outcomes of diverse aetiologies have contributed to interpretations of broad effects that cannot be explained entirely by improvements in glycaemic control or modest adverse effects of specific comparators (e.g. sulfonylureas and insulin) (Campbell et al., 2017; Foretz et al., 2023).

### 2.4. Better survival in type II diabetes than the general population

One of the most influential findings supporting metformin’s anti-aging potential came from a 2014 study by Bannister *et al.* titled ‘*Can people with type 2 diabetes live longer than those without? A comparison of mortality in people initiated with metformin or sulfonylurea monotherapy and matched, non-diabetic controls’* (Bannister et al., 2014). In this study, individuals with incident type II diabetes were stratified according to whether they initiated metformin monotherapy or sulfonylurea monotherapy treatment, and were compared to general population matched controls without diabetes (Bannister et al., 2014). Even without adjustment for additional comorbidities, metformin monotherapy initiators presented with marginally lower mortality overall, but with large effects in those aged 71–74 (Bannister et al., 2014). However, considerably higher mortality was observed in those initiating sulfonylureas, compared to general population controls. After adjustment for baseline cardiovascular prophylaxis, having type II diabetes and initiating on metformin monotherapy was associated with an 18 % higher median survival time compared to not having the disease at all (Bannister et al., 2014). The Emerging Risk Factor Collaboration studies suggest that a person with type II diabetes at age 50 has a reduced life expectancy of 6 years (Anisimov et al., 2005). Considering these risks, the survival differences reported in Bannister et al. (2014) suggest a substantial mortality reducing effect of metformin in persons with type II diabetes, with possible relevance to those without the disease as well (Campbell et al., 2017).

## 3. Recent developments on the anti-aging potential of metformin

### 3.1. Mixed evidence on biology of aging and lifespan in experimental models

The National Institute on Aging’s (NIA) Intervention Testing Program (ITP) is an experimental multi-centre peer-reviewed program that tests potential agents that may delay aging in mice, as measured by lifespan extension and/or delayed onset/severity of late life pathologies (Box 1) (Nadon et al., 2008, 2017; Macchiarini et al., 2021). The ITP reported in 2016 that metformin, administered to mice at a dose of 1000 milligrams per kilogram of food from 9 months of age, failed to produce significant lifespan benefits in either male (n = 442) or female (n = 421) mice (Strong et al., 2016). The ITP dose was chosen in an attempt to validate the findings by Martin-Montalvo et al. (2013) in which metformin was evaluated in mice of a single inbred genotype (C57BL/6). No benefit of metformin was observed in either sex or in any secondary analysis conducted by the ITP, including differential survival to the 90th percentile age of the joint survival distribution (Wang/Allison test), or across any of the three study sites analysed separately (Wang et al., 2004). A 2022 meta-analysis of experimental studies, including those from the ITP, similarly suggested that metformin was not associated with lifespan extension in mice (Parish and Swindell, 2022). These findings persisted across a range of subgroups, including when ITP data was not present in the analysis, suggesting this represents a broad pattern within the recent literature (Parish and Swindell, 2022).

Despite a lack of benefit from metformin monotherapy in the ITP, some evidence was provided suggestive of a synergistic effect when administered to rapamycin-treated mice. However, this effect was only statistically significant when using a non-protocol analysis excluding site stratification, and in males only (Strong et al., 2016; Lamming et al., 2013; Miller et al., 2011, 2014). Moreover, interpretation of these findings was complicated by the absence of a contemporaneous control cohort of mice given rapamycin alone, which necessitated comparisons with historical cohorts instead (Lamming et al., 2013; Miller et al., 2011, 2014). Promising findings have also recently been observed in primates. A small but comprehensive randomized study, in which 12 elderly male monkeys received metformin, suggested a significant slowing or reversal of a range of key biological aging indicators and improved performance across a battery of cognitive tests (Yang et al., 2024). However, no data was reported on lifespan, and pre-treatment measurements were not available for most aging biomarkers (pan-tissue transcriptomics, DNA methylomics, plasma proteomics and metabolomics) and functional memory outcomes assessed (Wisconsin General Test Apparatus) (Yang et al., 2024).

Most data on these biomarkers derive from observational cohort studies, where their predictive ability has been assessed with respect to various outcomes of aging (Moqri et al., 2024). However, even if such biomarkers are sensitive to interventions, for these changes to correspond to changes in functional, disease or lifespan endpoints, they must represent causal mediators within those relationships (Fleming and Powers, 2012). There is currently little evidence that these biomarkers behave in this way, and this cannot be assumed until formal validation has been done (Moqri et al., 2024). At a higher level, additional analytical validation is also needed to address difficulties with standardization across platforms and tissues, and internal statistical procedures (Moqri et al., 2024). For these reasons, aging biomarkers are not yet sufficiently validated as surrogate endpoints for their use in intervention studies and are currently best viewed as exploratory (Moqri et al., 2024).

### 3.2. Recognition of time-related and selection biases in observational studies

Were discrepancies arise between clinical trials and observational studies, such differences are often viewed through the lens of confounding, or equivalently, as a failure to emulate randomization (Dickerman et al., 2019). However, such distortions are often manageable, through either statistical adjustment, or careful consideration of their impact on the interpretation of data within a given study. Recent data suggest that appropriately designed observational studies can replicate the findings of randomized intervention trials, by for example, using target trial emulation and new-user pharmacoepidemiologic study designs (Wang et al., 2023a; Hernán et al., 2022; Johnson et al., 2013; Suissa et al., 2017; Lund et al., 2015; Wintzell et al., 2022). A major advantage of these approaches is that their implementation more explicitly prevent methodological errors that are frequently encountered in observational drug studies, namely “time-related biases” (Suissa and Dell’Aniello, 2020). These have considerable capacity for distorting associations of interest and are more difficult to diagnose and interpret than more well-known biases such as confounding (Suissa and Dell’Aniello, 2020; Suissa, 2008). Time-related biases have been particularly well-documented and severe in the observational literature comparing glucose-lowering therapies in type II diabetes (Suissa, 2017; Dai et al., 2022; Azoulay and Suissa, 2017; Patorno et al., 2015; Suissa and Azoulay, 2012; Bykov et al., 2019; Patorno et al., 2014). This constitutes a broad area of research from which much of the human evidence supporting the geroprotective potential of metformin has been derived.

Due to their structural nature, these sources of biases can produce an affected literature that systematically favours protective effects of newer treatments over older treatments, first-line treatments over second-line treatments, any treatment over no treatment, and higher over lower levels of cumulative use (see Box 2). Despite being an old drug, metformin entered mainstream use for type II diabetes in the late 1990s, succeeding its most common active comparators in observational studies, namely sulfonylureas and insulin, and did so primarily as a first-line agent (Higgins et al., 2016; Wilkinson et al., 2018; Cohen et al., 2003; Montvida et al., 2017). These historical facets have given metformin two systematic advantages in the face of studies affected by time-related biases. Given the presence and severity of these biases within the literature comparing glucose-lowering drugs, research summaries that do not consider their presence have serious limitations and warrant strong reservations as to the reliability of their conclusions, particularly when in search of interventional insight (Sterne et al., 2019; Egger et al., 1998; Li et al., 2021). For example, one of the most highly cited meta-analyses on the anti-aging effects of metformin in humans by Campbell et al. (2017) did not consider any source of bias beyond confounding, nor assess heterogeneity of results associated with their presence (Campbell et al., 2017).

A lesser-known issue commonly affecting observational drug studies relates to how control groups are defined when comparing exposure with non-exposure rather than use of an active comparator. New-user study designs and target trial emulation approaches correctly allow exposed individuals to serve as potential controls prior to their date of exposure (Wang et al., 2023a; Hernán et al., 2022; Johnson et al., 2013; Suissa et al., 2017; Lund et al., 2015; Wintzell et al., 2022). However, many studies have employed flawed designs where exposed individuals are compared only to those who never become exposed at any point during follow-up. This results in control groups comprised of only individuals who A) had insufficient person-time opportunity to become exposed or B) in which the exposure was contraindicated (Tran and Suissa, 2021; Lund et al., 2017). This artificially increases the risk of “terminal events” that prevent subsequent exposure, such as death. For a given outcome of interest, the magnitude of this bias scales positively with the cumulative incidence of exposure in the study population, duration of follow-up, and strength of the association between the outcome and death (Tran and Suissa, 2021; Lund et al., 2017). For people with type II diabetes, cumulative incidence of metformin use is usually extremely high, since it is the most commonly prescribed oral hypoglycaemic agent worldwide (Bailey and Turner, 1996). Therefore, studies comparing new-users and never-users of metformin within cohorts of type II diabetes are severely affected by this source of bias, even if other issues such as immortal time bias are sufficiently addressed.

Beyond the general account provided here, in subsequent sections we refer to the impact that time-related and other pharmacoepidemiologic biases have had on specific lines of research suggesting beneficial effects of metformin. However, even in the absence of such concerns, the vast majority of the observational literature on metformin has involved comparisons with users of other glucose-lowering drugs, either as active comparators or mixed ‘non-use’ of metformin. This complicates causal interpretations because several diabetes drugs, i.e. sulfonylureas and insulin, have long been suspected of increasing the risk of several adverse outcomes – most notably cardiovascular morbidity and all-cause mortality (Simpson et al., 2015; Khunti et al., 2018). Such increased risks are often only apparent in comparisons with other glucose-lowering drugs, as opposed to with placebo or lifestyle interventions, due to a net positive effect of hyperglycaemia prevention in the context of diabetes (Azoulay and Suissa, 2017; Monami et al., 2013b). Whilst recent progress has been made on understanding this issue, it is not yet a fully resolved matter, particularly for lesser studied outcomes that have not been subject to clinical trial investigations (Wexler, 2019). For these reasons, modest advantages of metformin compared to sulfonylureas or insulin in appropriately designed observational studies should be interpreted with caution.

### 3.3. No protective effect against cancer incidence or mortality

Observational studies reporting large reductions in cancer incidence and mortality have been instrumental in generating interest in repurposing metformin for its putative chemopreventive and chemotherapeutic effects (Pollak, 2014). Early meta-analyses reported reductions in risk of approximately of 30–40 %, for both cancer incidence and mortality (Decensi et al., 2010; Zhang et al., 2013; Noto et al., 2012). However, time-related biases have been shown to be highly prevalent in this literature (Suissa and Azoulay, 2012; Farmer et al., 2017). Suissa *et al.* summarized this phenomenon in 2012 and identified methodological flaws in a series of 24 influential studies on metformin and cancer (Suissa and Azoulay, 2012). Of these, 13 suffered from immortal time bias, 9 had not accounted for time-window bias, and 3 did not consider time-lagging issues when comparing first and second-line treatments (see Box 2) (Suissa and Azoulay, 2012). The reported effect sizes of these studies were shown to be compatible with their identified biases, which all favoured protective associations with metformin (Suissa and Azoulay, 2012). Meanwhile, appropriately designed studies at the time did not suggest that metformin was associated with reductions in cancer incidence or mortality compared to other glucose-lowering drugs (Azoulay et al., 2011; Smiechowski et al., 2013a, 2013b; Bensimon et al., 2014; Tsilidis et al., 2014; Kowall et al., 2014). Similarly, two meta-analyses of existing clinical trial data in type II diabetes also suggested no evidence of a protective effect of metformin against cancer incidence or mortality (Stevens et al., 2012; Thakkar et al., 2013).

Despite these early alarms, interest in the possible preventive or therapeutic effects of metformin persisted, and there are currently over 300 registered clinical trials assessing metformin in cancer treatment in the International Clinical Trials Registry Platform. The largest of these to be published to date was the highly anticipated MA.32 trial in high-risk invasive nonmetastatic breast cancer (Goodwin et al., 2022). In 3682 patients (mean age 52.4 years), metformin was not associated with improvements in overall (HR = 1.10) or progression-free survival (HR = 1.01) compared to placebo, over 5 years of follow-up (Goodwin et al., 2022). Other notable examples include the OCOG-ALMERA trial of locally advanced non-small lung cancer, and metformin in advanced pancreatic cancer, similarly reporting no evidence of benefit (Kordes et al., 2015; Tsakiridis et al., 2021). Metformin has thus far failed to demonstrate effectiveness as an adjuvant therapy across a wide range of cancer types, stages and prognoses (Yu and Suissa, 2023). The role of methodological flaws in the foundational literature inspiring these trials is often overlooked, and explanations for a lack of benefit are explored elsewhere (Foretz et al., 2023; Suissa, 2017; Yu and Suissa, 2023; Mortimer and Seewaldt, 2022; Gallagher et al., 2022; Cejuela et al., 2022; Lord and Harris, 2023).

A recent meta-analysis combined the results of 22 clinical trials using metformin as an adjuvant cancer therapy, including the MA.32 trial described previously. Across a total of 5943 participants, metformin was not associated with progression-free survival (HR = 0.97) or overall survival (HR = 0.98) for all cancers combined (Wen et al., 2022). When divided by cancer location, low certainty evidence suggested benefit in reproductive cancers (HR = 0.87) and deleterious effect in gastrointestinal cancers (HR = 1.45), although both findings may be due to random variability and type I error. An updated meta-analysis of clinical trials in type II diabetes and obesity provides more recent evidence, with even lower risk of type II error, that metformin is not associated with chemopreventive effects against cancer incidence in these disease states (Mesquita et al., 2024). Recent observational studies using appropriate methods similarly report no evidence of benefit (Dickerman et al., 2023; Dankner et al., 2019; Farmer et al., 2019). Further data on the prevention of cancer mortality in the context of prediabetes are reported in the long-term follow-up of the Diabetes Prevention Program (see below).

### 3.4. Little evidence for cardioprotective effects in high-risk populations

The use of metformin in type II diabetes has long been thought to have neutral or modest beneficial effects on a range of cardiovascular risk factors, including body weight, cholesterols, triglycerides, and blood pressure (Wulffelé et al., 2004; Tsapas et al., 2021; Gillani et al., 2021). Combined with long-term follow-up of its use in clinical trials supporting its safety, metformin has been firmly established as a preferred first-line agent in the treatment of type II diabetes (ElSayed et al., 2022). However, evidence for reductions in major cardiovascular events or mortality from intensive glucose control with metformin is mostly derived from a subgroup of a clinical trial conducted over 30 years ago (McCormack and Greenhalgh, 2000; Flory and Lipska, 2019; Boussageon et al., 2016). Although the randomized UKPDS reported large reductions in mortality and cardiovascular outcomes with metformin compared to dietary treatment, it also demonstrated equivalently large increases in these outcomes when metformin was added to sulfonylureas as combined therapy (UKPDS Research Group, 1998). This negative result has occasionally been omitted in analyses and interpretations of metformin’s cardiovascular and mortality effects, despite representing an equivalent quality of randomized evidence (McCormack and Greenhalgh, 2000; Boussageon et al., 2012). More recent meta-analyses of clinical trials of glucose-lowering medications have suggested that metformin may be neutral with respect to most cardiovascular event outcomes and mortality (Palmer et al., 2016; Zhu et al., 2020; Tsapas et al., 2020; Griffin et al., 2017). Evidence of benefit is therefore generally currently regarded as being uncertain - a point echoed by leading diabetes and cardiovascular associations (Davies et al., 2018; Marx et al., 2023; ACC/AHA, 2019).

Long-standing interest in the cardiovascular effects of metformin and identification of both glycaemic and non-glycaemic mechanisms has raised interest in its potential for repurposing outside of diabetes (Ferrannini and DeFronzo, 2015). Metformin has now been studied in several highly anticipated, heterogenous clinical trials assessing its cardiovascular effects in high-risk individuals without overt type II diabetes (Rena and Lang, 2018). None of these trials, except one, demonstrated an effect on their primary outcome. The CAMERA study assessed the role of metformin on the progression of atherosclerosis in nondiabetic patients (n = 173) with coronary artery disease, where no effect was observed on progression of mean distal carotid intima-media thickness (cIMT) (Preiss et al., 2014). The MetCAB trial (n = 111) assessed the effect of metformin on protection from myocardial injury during coronary artery bypass graft surgery and failed to demonstrate any benefit (Messaoudi et al., 2015). The GIPS-III trial (n = 380) assessed the effect of metformin on recovery from ST-segment elevation myocardial infarction, and demonstrated no effect on left ventricular ejection fraction or cardiovascular events (Lexis et al., 2014). The REMOVAL study in type I diabetes (n = 643) similarly failed to demonstrate an effect against the primary outcome mean cIMT, although an effect was observed on a tertiary outcome (Petrie et al., 2017). The smallest and most recent of these trials, MET-REMODEL (n = 68) demonstrated a benefit of metformin on change in left ventricular mass in prediabetic patients with coronary heart disease (Mohan et al., 2019).

There are several possible explanations for these largely negative findings. First, they may be compatible with metformin not having clinically meaningful cardiovascular effects beyond those dependant on reducing diabetic hyperglycaemia, although most patients in these trials had insulin resistance and/or prediabetes. This would also imply that metformin should be equivalent to other oral hypoglycaemic agents with neutral cardiovascular risk and similar glucose lowering effects. Another explanation implicates the modern environment of intensive cardiovascular risk management, which may impose a low ceiling effect on the cardiovascular benefits of metformin. Lastly, all of the trials assessing metformin’s cardiovascular effects outside of type II diabetes are relatively small and employed surrogate endpoints of cardiovascular function, which may limit their interpretation (Weintraub et al., 2015). However, interventions without effect on surrogate endpoints are rarely successful against cardiovascular events, and surrogate endpoint trials are much more effective at excluding rather than identifying patient benefit (Bikdeli et al., 2017). Further data on metformin’s effects against cardiovascular event endpoints in the context of prediabetes are reported in the long-term follow-up of the Diabetes Prevention Program (see below).

### 3.5. Long term follow-up of efficacy in prediabetes

The Diabetes Prevention Program (DPP) was a three-armed randomised placebo-controlled clinical trial designed to assess the effect of an intensive lifestyle intervention versus metformin versus placebo, in the prevention of type II diabetes (DPP Research Group, 2002). This landmark study of 3234 individuals with prediabetes demonstrated reductions in the incidence of diabetes by 58 % and 31 % with lifestyle intervention and metformin respectively compared to placebo, over a follow-up period of 2.8 years (DPP Research Group, 2002). Following premature termination of the initial trial period due to the success of the interventions, the DPP became the Diabetes Prevention Program Outcomes Study (DPPOS). In this follow-up study, all treatment groups were provided a modified lifestyle intervention program, and placebo was discontinued. However, the group initially randomized to metformin were instructed to continue receiving the study drug unmasked, in addition to the modified lifestyle program, until HbA1c rose above 7 %, at which point they received intensified or additional treatment for incident type II diabetes. Of all the original study participants, 88 % continued for follow-up in the DPPOS (DPPOS Research Group, 2015).

After 15 years of follow-up, cumulative diabetes rates in the original placebo, metformin and lifestyle arms of the study were 62%, 56%, and 55% respectively (DPPOS Research Group, 2015). However, annual diabetes incidence rates were similar in all three study arms throughout DPPOS follow-up, suggesting limited additional benefit of metformin in diabetes prevention on top of a modified lifestyle program (DPPOS Research Group, 2015). The DPPOS thus provides a valuable opportunity to assess metformin’s effects over a longer period of time, on outcomes relevant to its geroprotective potential. After 19–21 years of follow-up, 453 deaths had occurred in DPPOS participants, and metformin treatment was not associated with any differences in cardiovascular (HR = 1.08), cancer (HR = 1.04) or all-cause mortality (HR = 0.99) (Lee et al., 2021). Over the same timeframe, metformin was also not associated with any differences in the incidence of neither cancer (HR = 0.90, *p* = 0.31) nor major cardiovascular events (HR = 1.03), despite improvements in several cardiovascular risk factors (Goldberg et al., 2022; Orchard et al., 2005; Ratner et al., 2005; Goldberg et al., 2017; Heckman-Stoddard et al., 2025). These findings support the conclusions of trials assessing metformin’s specific effects against cancer and cardiovascular outcomes, described previously (Wen et al., 2022; Preiss et al., 2014; Messaoudi et al., 2015; Lexis et al., 2014; Petrie et al., 2017). However, out-of-study metformin use, declining adherence over a long follow-up period, and the modified lifestyle program all may have diluted observed effects in intention-to-treat analyses of the DPPOS (Gerstein, 2022).

Despite out-of-study metformin use due to incident diagnoses of diabetes in the original placebo arm, there was a wide gulf in the degree of its use across follow-up: 10.7 versus 2.3 metformin-years between 1996 and 2016 in the treatment and placebo groups respectively (DPPOS Research Group, 2015). However, the DPPOS investigators also provided additional analyses adjusting for carefully documented out-of-study metformin use, where all findings were highly robust to adjustment for this source of bias (Lee et al., 2021; Goldberg et al., 2022; Heckman-Stoddard et al., 2025; Luchsinger et al., 2017; Domalpally et al., 2023). More generally, declining adherence is a problem inherent to long-term trials of glucose-lowering drugs, and data for the DPPOS were similar to other trials in their comparable durations of follow-up (Walker et al., 2020). However, per-protocol analyses adjusting for metformin adherence similarly could not demonstrate statistically significant protective effects, where reported, despite being biased in favour of metformin due to selection (Lee et al., 2021). Lastly, interactions with the modified lifestyle program explaining the lack of observed benefit would suggest that metformin is susceptible to very modest ceiling effects, and only manifests its geroprotective effects in limited circumstances, if at all. The ongoing VA-IMPACT and MIMET trials may provide more definitive evidence of metformin’s possible cardiovascular and mortality effects, albeit in high-risk individuals with both prediabetes and established coronary artery disease (scheduled completion 2028–30) (Rena and Lang, 2018; Ritsinger et al., 2023; Dihoum et al., 2023).

### 3.6. Potential protection against severe COVID-19

The risk for severe COVID-19 infection and its complications increase with age and these outcomes have recently been incorporated into the geroscience framework as manifestations of aging-related disease processes (Barzilai et al., 2020; Justice et al., 2021). Factors underlying these patterns may include age-associated impairment of antiviral defences and dysregulation of monocyte and neutrophil inflammation in response to infection (Bartleson et al., 2021). Observational studies during the COVID-19 pandemic provided promising evidence on metformin’s association with severe infection and mortality in populations with type II diabetes (Ganesh and Randall, 2022). However, the design of observational drug studies on COVID-19 are highly susceptible to the time-related biases described previously, regardless if they are assessing preventative or therapeutic effects of candidate treatments (Franklin et al., 2021; Renoux et al., 2021). Notwithstanding, metformin has now been the subject of three major clinical trials assessing its effect on the progression from acute infection to severe COVID-19, in diverse populations with varying baseline risk according to age, BMI or multimorbidity.

In the first of these, the COVID-OUT trial, metformin reduced a tertiary outcome comprised of emergency room visits, hospitalization and death (HR = 0.62), but was without effect on its primary outcome (Bramante et al., 2022). Participants receiving metformin also showed reduced risk of long COVID-19 (HR = 0.59) after 10 months, partially explained by the lower occurrence of severe acute COVID-19 (Bramante et al., 2023). Metformin was most effective with increasing proximity to symptom onset and correlated with evidence of reduced viral load, potentially indicating an antiviral mechanism (Bramante et al., 2024; Ventura-López et al., 2022). In the subsequent TOGETHER trial, no effect of metformin was observed against any primary (Risk Ratio = 1.14) or secondary outcome studied (Reis et al., 2022). The most recent metformin trial (ACTIV-6) focused on symptomatology and recovery in mild-to-moderate COVID-19 during later stages of the pandemic, and reported no benefit against any primary (sustained recovery time) or secondary outcome (Bramante et al., 2025). However, findings on long-covid in the ACTIV-6 trial are yet to be reported, and may provide more scope for assessing evidence for benefit, given the low incidence of severe acute COVID-19 in this study population.

Attempts have been made to reconcile the mixed findings provided by these studies. First, COVID-OUT and ACTIV-6 employed instant-release metformin which was titrated to the target dose, while TOGETHER used extended-release metformin at the target dose without titration (Bramante et al., 2022; Reis et al., 2022). Second, TOGETHER did not exclude persons with type II diabetes and already taking metformin from being eligible for the trial. This led to inclusion of subjects using metformin in the control group and to potential double-dosing in the treatment arm (Expression of concern, 2024). A preliminary meta-analysis containing corrected data from TOGETHER and COVID-OUT suggests that metformin may be protective against developing severe COVID-19 infection in high-risk patients (Lee and Boulware, 2025). However, it is worth considering that approximately 50% of participants in the high risk COVID-OUT and TOGETHER trials were obese, and interpretations of geroprotective mechanisms here may be confounded by prediabetes and insulin resistance, for which metformin is already indicated (Bramante et al., 2022; Reis et al., 2022).

### 3.7. Unsuccessful replications of general population survival comparisons

Considerable shifts in risk factors are required to ameliorate differences in mortality and morbidity between people with type II diabetes and the general population (Tomic et al., 2022; Kaptoge et al., 2023; Wang et al., 2024). A landmark study suggested that only 5% of patients with type II diabetes achieved a sufficient risk factor profile that was associated with survival equal to that of the general population (Rawshani et al., 2018). From this perspective, it would be surprising if metformin monotherapy alone could compensate for such steep morbidity and provide equal, let alone better survival, than the general population, as suggested as by the influential Bannister et al. (2014) study, discussed previously (Bannister et al., 2014). However, the Bannister study had methodological features that may have created selection biases in favour of metformin. First, metformin initiators were censored if they intensified their diabetes treatment with additional medicines and so were only included in the study as long as they were doing well on monotherapy. Second, matched control groups were selected as being ‘never-diabetic’ by conditioning across the full range of future follow-up. This can potentially artificially increase mortality in the control group, as described in Section 3.2 (Tran and Suissa, 2021; Lund et al., 2017).

Two explicit replication studies have thus far failed to reproduce the findings demonstrated in Bannister et al. (2014). Keys, Hallas, Christensen et al. (2022) used the system of national registries in Denmark to compare mortality in persons with incident type II diabetes and general populations controls without diabetes (Keys et al., 2022). Four separate study designs were employed which allowed control for familial and genetic factors in discordant twin pairs, testing of metformin’s effects at different durations and levels of cumulative use, and assessing bias due to a range of methodological features discussed previously. The authors could not demonstrate a survival advantage in those initiating on metformin monotherapy, or even survival equalisation, compared to the background population, in any of their analyses. On the contrary, mortality was consistently increased compared to general population nondiabetic controls (HR = 1.48–2.05). Between the two studies, the only major difference was observed in the metformin monotherapy group’s mortality rates, where in the Bannister et al. (2014) cohort it was 43% lower than in Keys et al. (2022). Otherwise, mortality rates were identical for the sulfonylurea exposed, sulfonylurea controls, and metformin controls, as were a range parameters describing treatment trajectories and morbidity in all groups (see Box 3) (Keys et al., 2022). No epidemiological or methodological explanation could be determined for the differential findings across studies, despite extensive efforts (see Box 3).

The second replication study was published in 2023, this time extending the scope of follow-up to 20 years, compared to 5.5 years in Bannister et al. (2014), and 8 years in Keys et al. (2022) (Stevenson-Hoare et al., 2023). Using a robust time-dependent Cox regression approach, the authors reported a 69% and 59% increased hazard of death in metformin initiators and sulfonylurea initiators compared to matched controls without diabetes, over 20 years of follow-up. In a second matched cohort analysis, although demonstrating excess mortality for most of the follow-up period, the authors report survival equal to the background population for sulfonylurea initiators (Survival Time Ratio = 0.99), and a survival advantage for metformin initiators (STR = 1.21) in the first year of treatment (Stevenson-Hoare et al., 2023). However, the ratio of these two estimates is less than what is usually observed when directly comparing sulfonylureas versus metformin in appropriately designed observational studies (Azoulay and Suissa, 2017). As in Bannister et al. (2014), this matched cohort analysis also selected problematic ‘never-diabetic’ controls (Stevenson-Hoare et al., 2023). However, controls were additionally matched on baseline cardiovascular disease, of which 90% of patients were affected (Stevenson-Hoare et al., 2023). Since cardiovascular disease patients are at high risk of developing type II diabetes, use of never-diabetic controls here results in a selection bias that inflates mortality in the control groups, and is likely responsible for the modest first year effects of both drugs (Tran and Suissa, 2021; Lund et al., 2017).

### 3.8. Mixed evidence on the prevention and treatment of frailty

Three small clinical trials have assessed the short-term effects of metformin on functioning and quality of life in the context of prefrailty or frailty, with mixed results (Laksmi et al., 2017; Qaisar et al., 2024; Witham et al., 2025). In the first of these, nondiabetic prefrail individuals treated with metformin for 14 weeks reported increased gait speed, but no change in handgrip strength, serum myostatin, or health related quality of life (Laksmi et al., 2017). In the second, sarcopenic individuals received metformin for two months, and reported improvements in handgrip strength and sarcopenia-related quality of life, but no change in gait speed (Qaisar et al., 2024). In the most recent MET-PREVENT trial, metformin for 4 months did not improve grip strength, walking speed, physical performance, muscle mass, quality of life, or activities of daily living (Witham et al., 2025). These trials were all limited by poor retention of study participants, an issue inherent to studies of frail individuals. However, the MET-PREVENT trial was able to attain the highest retention rate thus far and reported that metformin was poorly tolerated by the study population (Witham et al., 2025).

Two small clinical trials have assessed the effect of metformin on exercise capacity and physiological adaptation in older adults. In first of these, treatment with metformin for 12 weeks resulted in an attenuation of exercise-induced improvements in whole-body insulin sensitivity, skeletal muscle mitochondrial respiration, and vo2 max after aerobic exercise (Konopka et al., 2019). In the second trial, treatment with metformin for 14 weeks parallel to a resistance training program resulted in no difference in exercise performance, and in smaller increases in lean mass gained by inhibiting the expected beneficial hypertrophic response (Walton et al., 2019). Lastly, the DPPOS assessed the prevalence of frailty at both 8- and 10-years post-randomization, and reported no preventative benefit or harm of metformin against this outcome (Hazuda et al., 2021). Overall, these preliminary findings suggest that metformin does not reliably prevent frailty or improve existing symptoms, and may even have adverse effects on physiological adaptation to exercise or be otherwise poorly tolerated in populations of older adults with frailty.

### 3.9. Preliminary evidence for other age-related outcomes

Metformin has also been investigated for its effects against dementia, age-related macular degeneration, osteoarthritis, osteoporosis, and hearing loss, albeit with less extensive scientific follow-up than other outcomes. In the case of dementia, meta-analyses of observational studies report mixed associations, and a familiar pattern has emerged with evidence of time-related biases contaminating the literature and appropriately designed studies tending to report no effect (Dai et al., 2022). However, pilot clinical trials have reported inconsistent but some positive evidence across a range of exploratory outcomes in various age-related cognitive syndromes (Hartman et al., 2019; Am et al., 2017; Lin et al., 2018; Luchsinger et al., 2016). In the DPPOS discussed previously, metformin had neither protective nor detrimental effect on a battery of in-person cognitive tests conducted 12 years after randomization, regardless of cumulative use (Luchsinger et al., 2017). The DPPOS is currently undertaking extensive phenotyping of cognitive syndromes in a cohort of 1800 persons, which will provide unique insight into the effects of very long-term metformin use on these conditions (Tahmi and Luchsinger, 2023). Three large randomized trials are also underway (MET-FINGER, MAP and MetMemory) to assess the effect of shorter term use of metformin on cognitive syndromes over approximately 3 years of follow-up (scheduled completion 20276–2029) (Tahmi and Luchsinger, 2023; Barbera et al., 2024).

Observational studies on metformin and age-related macular degeneration (AMD) have also led to conflicting findings (Elhalag et al., 2024; Holtz et al., 2023). However, comprehensive bias assessments of methodological factors in these studies, including time-related biases, have not yet been performed. Observational studies of AMD are also complicated by early-stage diagnoses often being incidental findings of asymptomatic disease, during routine eye tests performed for other reasons (e.g. in patients at high risk for diabetic retinopathy) (Chakravarthy et al., 2010). The DPPOS assessed this question using a cross sectional sample with clinically validated phenotyping of all participants, thus avoiding these limitations. Metformin was not associated with protection against AMD at any stage of the disease, regardless of cumulative use (Domalpally et al., 2023). The only available clinical trial, METforMIN (n = 66), reported no effect of metformin over 13 months on the progression of geographic atrophy, an advanced form of AMD (Shen et al., 2024). We are not aware of any further clinical trials planned to assess the effect of metformin on AMD.

Many observational studies comparing metformin to other glucose-lowering have generally reported favourable results for disease states such as osteoporosis and osteoarthritis, although systematic bias analyses of these literatures are absent (Bahardoust et al., 2023; Wang et al., 2023b). However, several small clinical trials in patients with knee osteoarthritis have consistently reported beneficial effects of metformin on inflammation and pain (Pan et al., 2025; Abed et al., 2024; Aiad et al., 2024; Alimoradi et al., 2023). On the other hand, metformin has been assessed for its anti-osteoporotic effects in a wide range of primary or secondary analyses of clinical trials for type II diabetes or polycystic ovary syndrome, and a recent meta-analysis thereof reported no evidence of benefit (Hu et al., 2023). Five observational studies have recently suggested that metformin may also be associated with lower risk of sudden hearing loss compared to other glucose-lowering drugs (Chen et al., 2019; Miwa et al., 2022; Huang et al., 2025; Tseng, 2023; Kim et al., 2023). However, as before, no systematic bias risk assessments of these studies have been conducted, and to the best of our knowledge, no clinical trials have evaluated the effect of metformin on hearing loss.

## 4. Questions Remain

There have been several important findings reporting in favour of metformin since its geroprotective potential was first raised. These include encouraging findings of protective effects in large clinical trials for COVID-19, pilot trials in cognitive syndromes and osteoarthritis, and analyses of multidimensional biological age and cognitive aging in elderly male monkeys. However, these promising developments need to be weighed up against an updated assessment of the contemporary experimental, observational and clinical trial data.

In terms of lifespan, metformin did not increase any relevant outcome in mice in the multi-center Intervention Testing Program, data from other experiments are mixed, and data for longer-lived mammals, including non-human primates, are not yet available. Replications of key observational studies initially supporting metformin’s geroprotective effects have generally not been successful. Various avoidable biases are widespread in the observational literature comparing glucose-lowering drugs which have, for historical reasons, been prone to systematically exaggerating protective effects of metformin in comparison to older drugs. Very few systematic reviews of observational studies have attempted to consider these well-documented issues, thereby under-mining an accurate interpretation of a literature that has otherwise been highly influential in generating support for metformin. Most observational studies have compared users of metformin to those of other glucose-lowering drugs, some of which have long been suspected of modestly increasing adverse outcomes in and of themselves after taking into account beneficial effects of hyperglycaemia prevention, further complicating causal interpretations. Even within metformin’s indication for type II diabetes, its often-presumed long-term cardiovascular benefits are also not entirely certain – a point echoed by leading European and American diabetes and cardiovascular associations.

Most importantly however, metformin has so far not demonstrated its anticipated preventative or therapeutic effects in most, although not all, major clinical trials in nondiabetic or prediabetic populations conducted to date. These trials provide important insight into its potential for translational success outside of type II diabetes, with respect to several important sequela of aging, including frailty, cancer, cardiovascular disease and mortality, all of which are key outcomes for currently planned clinical trials in the geroscience field. The results of ongoing clinical trials assessing the effect of metformin in cognitive syndromes and prediabetes with established cardiovascular disease are highly anticipated, given the encouraging pilot and experimental data supporting their rationale. Indeed, metformin’s place in the targeted treatment of high-risk populations will be determined on the basis of the evidence from these trials. However, for ongoing discourse within the geroscience community, their potential influence may be limited by their distant completion dates, which may fall after the imminent decision-making periods most relevant to contemporary metformin proposals.

For a large-scale intervention trial aiming to demonstrate its geroprotective effects against a broad spectrum of diseases, metformin originally offered a high likelihood of success due to a wide range of promising data at the time of its ascent. Whether metformin still occupies this position today is an open and increasingly relevant question, given the translational movement unfolding within the geroscience field, and the consequences and opportunity costs of its first randomized double-blind phase III trials (López-Otín et al., 2023).

## Data Availability

No data was used for the research described in the article.

## Box 1 Methodology, findings and implications of the Interventions Testing Program.

### NIA’s Interventions Testing Program

The National Institute on Aging (NIA) established the Interventions Testing Program (ITP) in 2004 to systematically assess the effects of dietary or pharmacological interventions on lifespan in mice.

#### Methodology

1. Three study sites contribute data and to its analysis, with protocols to protect against site-to-site variation.
2. Genetically heterogeneous cohorts of UM-HET3 mice are employed to enhance the translational potential of the findings.
3. Pilot studies are conducted to maximize the chances of success. These include tests to determine compounds’ stability in food, bioavailability, bioactivity, and in some cases, toxicity.
4. The employed sample sizes are sufficient to detect changes in mean lifespan of 10% with statistical power of at least 80%, using data from only 2 out of 3 study sites.

#### Findings

As of 2024, 12 pharmacological interventions have demonstrated statistically significant improvements in median lifespan in at least one sex.

- **Males and females**: Rapamycin, Acarbose, Glycine
- **Males only**: Aspirin, 17aEstradiol, Nordihydroguaiaretic acid, Protandim, Canagliflozin, Astaxanthin, Meclizine, 16-alpha-hydroxyestradiol
- **Females only**: Captopril

#### Implications

1. Manipulating lifespan with pharmacological intervention is feasible and facilitates functional improvements in healthspan.
2. Complex and disparate mechanisms underlie these improvements and indicate a broad scope of biological targets for modulating aging.
3. Marked sex differences in response to the tested interventions highlight the importance of their broad consideration in aging research.

## Box 2 Time-related biases in pharmacoepidemiologic studies of glucose-lowering drugs.

### Time-related biases in pharmacoepidemiologic studies of glucose-lowering drugs

#### Immortal time bias

In cohort studies, immortal time bias occurs when the time between cohort entry and drug exposure is included as part of follow-up. By definition, these periods are “immortal” (if outcome is survival) or “disease-free” (if outcome is disease incidence) and artificially inflate the proportion of follow-up spent alive or disease-free.

In comparisons of glucose-lowering drugs, this bias increasingly favors more recently adopted treatments (e.g., metformin versus sulfonylureas or insulin), as they have more “immortal” follow-up time between cohort entry and their initiation. Initiating follow-up from the time of exposure prevents this bias.

#### Time window bias

In case-control studies, this bias occurs when there are differential windows of opportunities for exposure between those experiencing the disease outcome of interest (cases) and not (controls). Relatively shorter windows of opportunity lead to an overrepresentation of unexposed cases and thus favor the exposure.

In comparisons of glucose-lowering drugs, this bias favors newer versus older treatments (e.g., metformin versus sulfonylureas or insulin). Time-window bias also favors higher levels of cumulative use, due to the increasing windows of opportunity needed to accumulate a larger number of prescriptions. Matching on cohort entry and index date (arrow 1) ensures equal exposure opportunity and prevents this bias.

#### Time lag and latency bias

In cohort studies, time lag and latency biases can arise in comparisons of first-line and second-line therapies when not taking into account treatment and disease history.

In comparisons of glucose-lowering drugs, incorrect analyses comparing incident initiators of one drug with prevalent users of another (arrow 1) may misattribute adverse effects of cumulative diabetes exposure to the initiation of second-line drugs and artificially favor comparisons with the first-line drug (e.g., metformin as first-line versus sulfonylureas or insulin as second-line). Matching follow-up on diabetes duration (arrow 2) prevents these biases.

#### Other time-related biases

A range of other time-related biases exist that are relevant to discussions of glucose-lowering drugs. These include reverse causality, and selection biases due to never-exposed controls, prevalent users, and informative censoring. However, the direction of these biases often depend on a myriad of factors specific to individual studies and are described throughout the rest of the review where relevant.

## Box 3 Comparisons of studies assessing the survival of persons with type II diabetes and the general population.

### Comparison of studies assessing survival in type II diabetes treated with metformin

Keys et al. (2022) reported that individuals with type II diabetes exhibit higher mortality than the general population (HR = 1.48). These findings contrasted with the highly influential study by Bannister et al. (2014), in which a survival advantage was reported. However, no methodological or epidemiological explanation could be identified for these differential findings, despite extensive efforts.

#### Comparisons across study cohorts

The data provided by Keys et al. facilitate a direct comparison with Bannister et al. across key outcomes and epidemiological parameters. Metformin initiators across both studies were very similar, including age at baseline, sex, duration of metformin monotherapy, and prevalence of lipid-lowering, anti-hypertensive, and anti-platelet medication at baseline.

However, mortality in metformin initiators was 43% lower in Bannister et al. than Keys et al. This was despite parity of mortality across all other groups, including metformin matched controls, sulfonylurea initiators, and sulfonylurea matched controls. These patterns suggest that the differential metformin findings between the two studies are unlikely to be the result of health differences in the matched controls, or systematic study design factors leading to a lack of replication across all analyses.

#### Methodological factors

In Bannister et al. (2014) metformin initiators were censored if they changed or intensified treatment, which is likely informative due to its association with diabetes progression and its complications. Keys et al. (2022) tested the impact of this in their data, reporting an attenuated hazard ratio of 1.39. Additional bias analyses similarly reported modest or no differences. None of the sensitivity analyses conducted could explain the extent of the contrast in metformin survival between the two studies.

**Table:**

Characteristic/Outcome	Keys et al. (2022)	Bannister et al. (2014)
Age at baseline (metformin group)	60.1 years	61.2 years
Duration of monotherapy (metformin group)	3.0 years	3.0 years
Proportion of men (metformin group)	53%	57%
Prior lipid-lowering therapy (metformin group)	46%	50%
Prior antihypertensive therapy (metformin group)	64%	66%
Prior antiplatelet therapy (metformin group)	31%	36%
Crude mortality rate / 100,000 py (metformin group)	24.9	14.4
Crude mortality rate / 100,000 py (sulfonylurea group)	49.0	50.9
Crude mortality rate / 100,000 py (metformin controls)	16.9	15.2
Crude mortality rate / 100,000 py (sulfonylurea controls)	28.4	28.7
